# The effects of olanzapine on genome-wide DNA methylation in the hippocampus and cerebellum

**DOI:** 10.1186/1868-7083-6-1

**Published:** 2014-01-02

**Authors:** Melkaye G Melka, Benjamin I Laufer, Patrick McDonald, Christina A Castellani, Nagalingam Rajakumar, Richard O’Reilly, Shiva M Singh

**Affiliations:** 1 Molecular Genetics Unit, Department of Biology, The University of Western Ontario, London, ON N6A 5B7, Canada; 2 Department of Psychiatry, The University of Western Ontario, London, ON N6A 5B7, Canada; 3 Department of Neuroscience, The University of Western Ontario, London, ON N6A 5B7, Canada; 4 Children's Health Research Institute, 800 Commissioners Road East, London, ON N6C 2V5, Canada

**Keywords:** Epigenetic, Hippocampus, Cerebellum, DNA methylation, Psychosis, Rat

## Abstract

**Background:**

The mechanism of action of olanzapine in treating schizophrenia is not clear. This research reports the effects of a therapeutic equivalent treatment of olanzapine on DNA methylation in a rat model *in vivo*.

Genome-wide DNA methylation was assessed using a MeDIP-chip analysis. All methylated DNA immunoprecipitation (MeDIP), sample labelling, hybridization and processing were performed by Arraystar Inc (Rockville, MD, USA). The identified gene promoters showing significant alterations to DNA methylation were then subjected to Ingenuity Pathway Analysis (Ingenuity System Inc, CA, USA).

**Results:**

The results show that olanzapine causes an increase in methylation in 1,140, 1,294 and 1,313 genes and a decrease in methylation in 633, 565 and 532 genes in the hippocampus, cerebellum and liver, respectively. Most genes affected are tissue specific. Only 41 affected genes (approximately 3%) showed an increase and no gene showed a decrease in methylation in all three tissues. Further, the two brain regions shared 123 affected genes (approximately 10%). The affected genes are enriched in pathways affecting dopamine signalling, molecular transport, nervous system development and functions in the hippocampus; ephrin receptor signalling and synaptic long-term potentiation in the cerebellum; and tissue morphology, cellular assembly and organization in the liver. Also, the affected genes included those previously implicated in psychosis.

**Conclusions:**

The known functions of affected genes suggest that the observed epigenetic changes may underlie the amelioration of symptoms as well as accounting for certain adverse effects including the metabolic syndrome. The results give insights into the mechanism of action of olanzapine, therapeutic effects and the side effects of antipsychotics.

## Background

Schizophrenia is one of the most devastating of psychiatric disorders [1]. The treatment of schizophrenia requires the suppression of hallucinations, delusions, agitation and the behavioural problems that accompany these symptoms [2]. Psychotherapy and rehabilitation can be undertaken when the acute symptoms start to subside through antipsychotic drug treatment.

The first antipsychotic drug, chlorpromazine, introduced in the early 1950s, was a major breakthrough because, unlike previously used sedative drugs, it could ameliorate hallucinations and delusions without overly sedating the patient [3]. Many other antipsychotic drugs were subsequently introduced [4], but these have not significantly advanced the treatment of schizophrenia. The early promise of the second-generation antipsychotics (atypical antipsychotics), such as clozapine and olanzapine, has been replaced by an acceptance that they are no more effective than the first-generation drugs [5]. However, second-generation antipsychotics have recently shown positive effects on verbal cognition [6]. Second-generation drugs have fewer neurological side effects but, unfortunately, many induce weight gain and the metabolic syndrome [7-10].

Our current understanding of the cause of schizophrenia is based on the pharmacological effects of the antipsychotic drugs used to treat the illness: they all bind to post-synaptic dopamine receptors especially D2 and the affinity at D2 receptors is both necessary and sufficient for the antipsychotic effects [11]. This, coupled with the observation that drugs that release dopamine into the synaptic cleft can induce the positive symptoms of schizophrenia (behaviours and feelings that are not real but imaginary), led to the hypothesis that excessive dopamine transmission in certain brain regions may cause the symptoms of schizophrenia [12]. A post-synaptic blockade occurs rapidly after a person ingests an antipsychotic drug. In contrast, the therapeutic effects of antipsychotics take days or weeks to accrue [13]. This suggests that downstream effects are important. One possibility is that the post-synaptic dopamine blockade causes a downstream cascade that has a therapeutic effect through altered gene transcription [14,15]. A downstream effect, such as altered transcription, would explain the delay in the onset of therapeutic action. Other clinical observations also demonstrate the need for a more complex model than a post-synaptic dopamine blockade. Patients frequently fail to respond to an antipsychotic but subsequently show a robust response to a different drug despite the fact that both block the D2 receptor [16]. Moreover, many patients with schizophrenia show only a partial response to antipsychotics or fail to respond at all [17]. A refinement of the dopamine hypothesis proposes that an increase of D2 levels in the striatum may cause hallucinations and delusions and reduced D1 levels in the frontal lobes may cause cognitive deficits [18,19]. This model is compatible with the delayed treatment effect but cannot explain the individual responsiveness to antipsychotics.

Epigenetic changes are another mechanism used to explain these clinical observations. They also offer an alternative therapeutic target for this serious disease: after 50 years of frustration we need to move our focus beyond post-synaptic dopamine receptors. Epigenetic changes associated with a drug can alter the expression of a single or variable number of genes without altering the gene sequence(s) [20]. Specifically, DNA methylation is a core epigenetic mechanism that involves the covalent binding of a methyl group to the 5-carbon position of cytosine leading to altered gene expression [21]. It is influenced by stochastic events including exposure to a variety of environments such as drug treatment [22,23]. If DNA methylation plays a role in drug response, the drug or its metabolite must modify the methylation profile of the genome [20].

Limited research demonstrates that antipsychotic drugs can alter DNA methylation and gene expression [24]. However, most of this research has been conducted using variable post-mortem human brain tissues [25] and inappropriate non-brain cell types [26] that are not always ideal. Therefore, we have assessed the effects of a therapeutic equivalent dose of olanzapine (2.5 mg/kg per day for 21 days), a commonly used antipsychotic, on DNA methylation in rat brains using rat methylation arrays. The results demonstrate for the first time that the effect of olanzapine on DNA methylation is widespread and tissue specific, which may account for its efficacy and adverse effects.

## Results

First, we assessed the locomotor activity of rats split into olanzapine-treated and vehicle-treated groups. Activity was significantly decreased (*P* = 0.001) in the olanzapine-treated group compared to the vehicle-treated group (Additional file 1: Figure S1a). Further, olanzapine-treated rats significantly increased in weight (*P* = 0.004) compared to the control group (Additional file 1: Figure S1b). Second, we assessed gene-specific DNA methylation across (almost) all genes in response to olanzapine treatment on two brain regions (hippocampus and cerebellum) and the liver, as detailed below.

### Olanzapine causes widespread and tissue-specific changes in genome-wide methylation in rats *in vivo*

**Figure 1:**
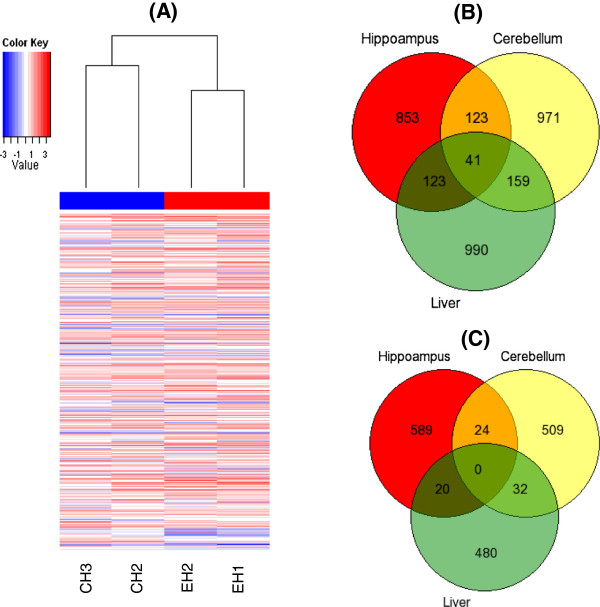
**Heat map of differential DNA methylation and the number of genes showing a) increased and b) decreased methylation. (A)** Heat map of differential DNA methylation enrichment peaks of genes in the hippocampus, following olanzapine treatment of rats. The average normalized log_2_-ratio scan values were used to calculate the M' value (M' = Average(log_2_MeDIPE/InputE) – Average(log_2_MeDIPC/InputC)) for each probe. Venn diagrams depicting the number of genes that show an increase **(B)** or decrease **(C)** in methylation in brain tissues and the liver, following olanzapine treatment of rats.

Widespread changes in gene-specific DNA methylation were apparent in all three tissues studied (hippocampus, cerebellum and liver), as shown by the heat map for the hippocampus (Figure 1A). The results identified genes where there was an increase or a decrease in methylation in drug-treated rats compared to controls. Specifically, almost twice as many genes showed an increase compared to the genes that showed a decrease in methylation, in response to olanzapine in each of the three tissues (Additional file 2: Table S1A, Additional file 3: Table S1B, Additional file 4: Table S2A, Additional file 5: Table S2B, Additional file 6: Table S3A and Additional file 7: Table S3B). Also, the set of genes affected differs across the three tissues. Approximately 75% of genes with an increase (Figure 1B) and over 90% of the genes with a decrease (Figure 1C) in methylation, following olanzapine treatment, were specific to a given tissue. Further, there was a small number of genes with a similar pattern of increase (total 164) or decrease (total 24) in the two brain regions and a smaller number with an increase (41) in all three tissues. The tissue-specific results are novel and were further assessed as follows.

### Pathways and associated network functions of genes that had changes in methylation in the hippocampus following olanzapine treatment

The genes (Additional file 2: Table S1A and Additional file 3: Table S1B) that had an increase (Table 1a) or a decrease (Table 1b) in methylation in the rat hippocampus following olanzapine treatment were assessed by pathway analysis. Genes with increased methylation were predominantly enriched in the dopamine-DARPP32 feedback in cAMP signalling canonical pathway (*P* = 1.6 × 10^–3^). The associated network functions that were affected included metabolic diseases and neurological disorders (Table 1, Additional file 1: Figure S2). In addition to the changes caused by olanzapine in methylation in psychosis-related canonical pathways, the results showed decreased methylation of genes involved in CDC42 and calcium signalling (*P* = 2.5 × 10^–3^) in the hippocampus. These genes affect nervous system development and function. Also, the cellular effects of sildenafil (Viagra) were revealed as an interesting canonical pathway (*P* = 9.8 × 10^–3^) for the 123 genes that had increased methylation as a result of olanzapine treatment in the two brain regions (Additional file 1: Figure S3).

**Table 1:** Top pathways and associated networks identified by pathway analysis for the hippocampus following olanzapine treatment

**(a) Top canonical pathways (Genes with increased methylation)**	***P-*** **value**	**No of molecules**^**a**^
Dopamine-DARPP32 feedback in cAMP signalling	1.65 × 10^–3^	20/157 (0.127)
CD27 signalling in lymphocytes	2.42 × 10^–4^	11/54 (0.204)
Oestrogen-mediated S-phase entry	2.56 × 10^–3^	6/26 (0.231)
Role of JAK2 in hormone-like cytokine signalling	3.38 × 10^–3^	7/34 (0.206)
**Associated network functions**		
Metabolic disease, endocrine system and developmental disorders		35
Cell cycle, cellular growth and proliferation, cell death		24
Molecular transport, neurological disease, cell-to-cell signalling		10
**(b) Top canonical pathways (Genes with decreased methylation)**	***P-*** **value**	**No of molecules**
CDC42 signalling	2.52 × 10^–3^	11/131 (0.084)
Prostanoid biosynthesis	2.55 × 10^–3^	3/9 (0.333)
Calcium signalling	5.92 × 10^–3^	12/178 (0.067)
D-myo-inositol (1,3,4,5,6)-tetrakisphosphatebiosynthesis	6.18 × 10^–3^	8/48 (0.167)
**Associated network functions**		
Developmental disorder, cell death and survival, cellular development		12
Molecular transport, nervous system development and function		10
Carbohydrate metabolism, cell morphology, lipid metabolism		9
Cellular development, skeletal, muscular and cardiovascular system development and function		8

^a^For the top canonical pathways, the ratio is the number of molecules in a given pathway that meet the cut-off (*P* ≤ 0.01) divided by the total number of molecules in the pathway.

### Pathways and associated network functions of genes that had changes in methylation in the cerebellum following olanzapine treatment

**Figure 2:**
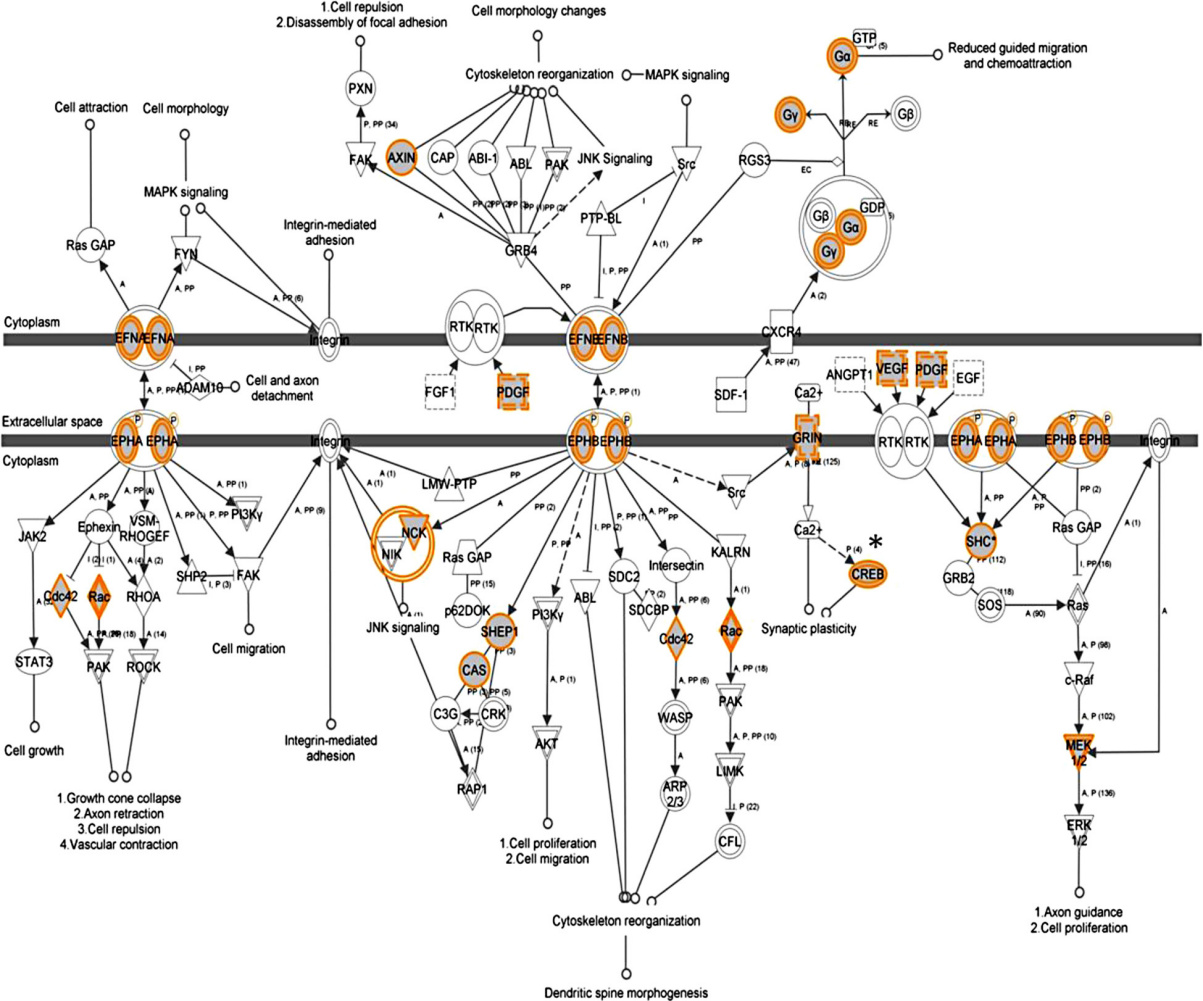
**Ephrin receptor signalling is the most significant canonical pathway in an olanzapine-treated cerebellum.** An asterisk indicates a gene previously implicated in psychosis (from Ingenuity Pathway Analysis).

We also analysed genes that had increased methylation in the cerebellum (Additional file 4: Table S2A and Additional file 5: Table S2B). The most significant pathway identified for the cerebellum was for ephrin receptor signalling (*P* = 5.23 × 10^–4^) (Table 2a; Figure 2). Synaptic long-term potentiation (*P* = 2.94 × 10^–3^), which is implicated in learning and plasticity, was among the most significant pathways identified (Additional file 1: Figure S4). Moreover, pathways involved in signalling (Erk/Mapk, circadian rhythm and protein kinase A) were identified (Table 2a). Interestingly, genes with reduced methylation were also involved in pathways such as ephrin B signalling (Table 2b).

**Table 2:** Top networks identified by pathway analysis for the cerebellum following olanzapine treatment

**(a) Top canonical pathways (Genes with increased methylation)**	***P-*** **value**	**No of molecules**^**a**^
Ephrin receptor signalling	5.23 × 10^–4^	24/176 (0.136)
Erk/Mapk signalling	1.59 × 10^–3^	24/184 (0.130)
Circadian rhythm signalling	1.94 × 10^–3^	8/33 (0.242)
Protein kinase A signalling	2.61 × 10^–3^	41/372 (0.110)
Synaptic long-term potentiation	2.94 × 10^–3^	17/113 (0.150)
**Associated network functions**		
Cardiovascular disease, cell signalling, small molecule biochemistry		25
Cellular development, tissue morphology, cardiac dilation		23
Molecular transport, protein synthesis, protein trafficking		12
Behaviour, nervous system development and function		11
Neurological disease, psychological disorders, cell-to-cell signalling		10
**(b) Top canonical pathways (Genes with decreased methylation)**	***P-*** **value**	**No of molecules**
Ephrin B signalling	4.0 × 10^–3^	7/72 (0.097)
G beta gamma signalling	4.1 × 10^–3^	8/99 (0.081)
Germ cell-Sertoli cell junction signalling	5.0 × 10^–3^	11/148 (0.074)
tRNA splicing	8.3 × 10^–3^	4/32 (0.125)
Tetrahydrofolate salvage from 5, 10 methenyltetrahydrofolate	8.6 × 10^–3^	2/6 (0.333)
**Associated network functions**		
Cell death and survival, cellular development		14
Energy production, lipid metabolism, small molecule biochemistry		14
DNA replication and repair, development, carbohydrate metabolism		14
Neurological disease, cellular function and maintenance, molecular transport		11

^a^For the top canonical pathways, the ratio is the number of molecules in a given pathway that meet the cut-off (*P* ≤ 0.01) divided by the total number of molecules in the pathway.

### Pathways and associated network functions of genes that had changes in methylation in the liver following olanzapine treatment

The results from a non-brain tissue sample also showed that the effects of olanzapine are not restricted to brain regions; it may also affect liver the (Additional file 6: Table S3A and Additional file 7: Table S3B). Genes that had an increase (Additional file 8: Table S4a) or a decrease (Additional file 8: Table S4b) in methylation in the liver, were in several pathways including lipid metabolism, cell death and organ morphology. Such genes include *DRD1/DRD2*, *NMDAR* and *PTEN* (Additional file 1: Figure S5).

## Discussion

There are a number of methods to test the effects of antipsychotic drugs, including the locomotor activity test [27] and the prepulse inhibition test [28]. In this study, we used the locomotor activity test. Significantly reduced locomotor activity in olanzapine-treated rats in this experiment (Figure 1A,B) has suggested that the drug administration paradigm employed was sufficient to cause therapeutically relevant effects in rats. Comparable therapeutic doses in rats were effective in previous studies and resulted in locomotor-suppressive effects [27]. Moreover, the significant increase in weight of olanzapine-treated rats in this and previous studies [29] indicated that the paradigm adapted might also be capable of causing metabolic disturbances, as seen in patients taking olanzapine for a long time [30]. Interestingly, the molecular results showed that olanzapine treatment caused genome-wide DNA methylation changes (Figure 1). Further, the results showed that most genes affected were tissue specific (hippocampus, cerebellum or liver). Also, the gene-specific methylation changes affected a number of networks that were tissue-specific, as expected. More importantly, the identified networks support two known effects of olanzapine, discussed in the following sections. The first is the recovery from psychosis [31] and the second is the adverse effects [32] of olanzapine. Olanzapine-induced DNA methylation changes in genes involved in canonical pathways may alter the associated network functions. However, further study is required to analyse the effects (on a protein level) of, specifically, the gene-specific methylation changes on each identified network.

We argue that the two manifestations could be attributed to tissue-specific alterations that disturb the coordinated expression of genes critical in the identified networks (Tables 1 and 2). This model is backed by a number of observations. First, the phenotypic effect of olanzapine is not immediate; rather it takes days or weeks after the initiation of treatment [13]. This may be the time that is needed for gene-specific methylation to alter the expression of the specific genes [33,34]. Also, patients may not respond to this drug, depending on their *CYP 1A2* genotype, which can metabolize this drug [35] or acquire resistance. Patients may need to take a different type of antipsychotic drug [16]. During this time, a patient may be affected by metabolic disorders, weight gain and related adverse effects [30]. We will discuss the specific mechanisms of the effects of olanzapine in the following section.

### Olanzapine-based psychosis recovery may involve changes in gene methylation

We argue that an increase or a decrease in methylation of specific gene promoters, following olanzapine treatment, may decrease or increase their transcriptional efficiency [36,37], specifically for the hippocampus, which is one of the primary sites responsible for psychotic symptoms [15,38,39]. Further, the pattern of transcriptional efficiency may also be modulated by other factors such as chromatin structure and elongation efficiency [38,40,41]. We acknowledge that the prefrontal cortex and nucleus accumbens, which are also implicated in psychosis [40,42,43], may need to be investigated in future studies.

**Figure 3:**
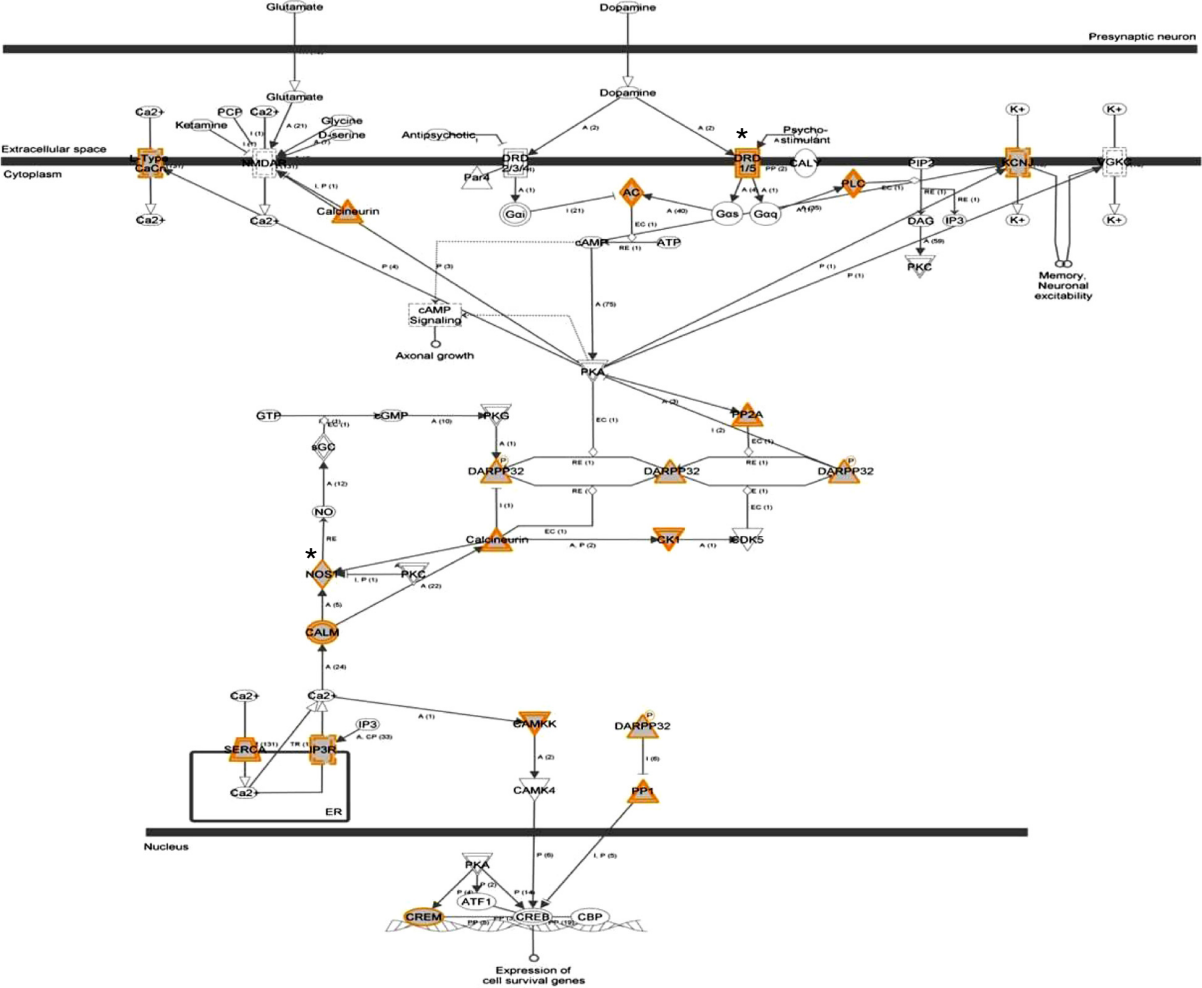
**Dopamine-DARPP32 feedback in cAMP signalling is a significant canonical pathway in an olanzapine-treated hippocampus.** An asterisk indicates a gene previously implicated in schizophrenia (from Ingenuity Pathway Analysis).

We note that in the hippocampus, dopamine-DARPP32 feedback in the cAMP signalling pathway (*P* = 1.6 × 10^–3^) was the most significant pathway identified. Neurons in the midbrain release dopamine, which modulates cAMP (cyclic adenosine 3,5-monophosphate) production by activating dopamine receptors [44]. These results suggest that the antipsychotic effects of olanzapine may involve alterations in gene-specific methylation that leads to the dysregulation of genes involved in dopamine-DARPP32 feedback in the cAMP signalling pathway (Figure 3). This includes several differentially methylated genes such as *Drd1/5* and *Nos1*. The dopamine blockade leads to the progressive reduction of psychosis while its disturbance leads to psychosis [45]. All antipsychotics block post-synaptic D2 receptors [11]. A serotonin-dopamine antagonist was formulated following the synthesis of second-generation antipsychotics [11]. However, patients frequently fail to respond to one antipsychotic but respond to a different drug even if both block the D2 receptor [16]. Also, schizophrenia patients may partially respond to an antipsychotic or do not respond at all [17]. This may be due to several factors, and one possibility would be the delay in the onset of therapeutic actions partly or fully influenced by the downstream effects, such as altered transcription [14,33]. As such, the differentially methylated genes involved in the dopamine-signalling pathway may stop or reduce transcription and gene expression [14,21,33]. In fact, decreased expression of *DARPP32* in the prefrontal cortex has been reported in schizophrenia patients [43,46,47]. Also, DNA methylation differences have been observed in the dopamine D2 receptor gene within and between pairs of monozygotic twins discordant for psychoses [48] and there is an overwhelming evidence for the involvement of dopamine in psychosis including schizophrenia [19].

Further studies on the effects of drugs may help to identify the genes and pathways that underlie psychosis. For example, a decreased expression of *CDC42* was reported in the cerebral cortex of schizophrenic patients in post-mortem studies, and this has been implicated in defects in dendritic spines in cortical neurons in the patients [49]. *CDC42* can reorganize septin fibre formation, which is thought to stabilize actin filaments needed for a normal spine shape and synaptic plasticity, as reviewed in Ide and Lewis [49]. However, cautious interpretation of the results is important because actual epigenetic changes in schizophrenic patients may represent changes in methylation status [50].

Our results show that olanzapine caused an increase or a decrease in the methylation of genes previously implicated in schizophrenia (Table 3), which may reflect the fact that olanzapine could alleviate psychiatric symptoms via mechanisms involving DNA methylation. Among the genes that decreased in methylation in the hippocampus is *Map6*, which is implicated in schizophrenia [51] and is involved in molecular transport, nervous system development and function (Additional file 1: Figure S6). This emphasizes that methylation may serve an intermediary role whose actual effect is realized through gene expression.

**Table 3:** Genes positively associated with schizophrenia where methylation changed in response to olanzapine

**Gene**^**a**^	**Reference (PubMedID)**	**Affected in**	**Peak length (bp)**	**Peak to transcription start site (bp)**	**Promoter class**
*Nos1*	20645313	Hippocampus	334	-3,719	LCP
*Map6*	16624526	Hippocampus	169	-2,382	ICP
*Drd5*	11304828	Hippocampus	844	41	HCP
*Cacna1c*	23183239	Hippocampus	425	-3,692	LCP
*Bdnf*	16818862	Hippocampus	329	-492	ICP
*Gsksb*	18500637	Hippocampus	573	-1,042	HCP
*Tf*	18045615	Hippocampus	159	-3,020	LCP
*Cnp*	16389193	Hippocampus	464	1,198	HCP
*Amacr*	20875727	Hippocampus	558	164	HCP
*Psen2*	19232479	Hippocampus	249	620	LCP
*Pax6*	10376119	Hippocampus	341	326	ICP
*Dlx1*	18384059	Hippocampus	431	-2,043	HCP
*Dlx1*	18384059	Cerebellum	1,330	257	HCP
*Cnp*	16389193	Cerebellum	769	940	HCP
*Psen2*	19232479	Cerebellum	455	442	LCP
*Pax6*	10376119	Cerebellum	349	-3,481	ICP
*Stx1a*	15219469	Cerebellum	149	72	HCP
*Drd5*	11304828	Cerebellum	264	-169	HCP
*Mmp9*	20037727	Cerebellum	138	-325	LCP
*Tnf*	11244489	Cerebellum	269	49	LCP
*Grin1*	12679240	Cerebellum	264	-1,595	HCP
*Tf*	18045615	Cerebellum	749	-1,704	LCP
*Dlg4*	21151988	Cerebellum	134	-1,459	LCP
*Amacr*	20875727	Cerebellum	138	-48	HCP
*Dao*	19685198	Cerebellum	287	-2,385	LCP
*Dlx1*	18384059	Liver	171	-2,993	HCP
*Cnp*	16389193	Liver	644	-2,644	HCP
*Sod2*	15193990	Liver	550	594	HCP
*Drd5*	11304828	Liver	369	-316	HCP
*Il6*	20393813	Liver	789	-3,030	LCP
*Comt*	11381111	Liver	638	-3,542	HCP
*Apoe*	14674716	Liver	2,158	-1,223	LCP
*Drd2*	18829695	Liver	257	-91	HCP
*Nos1*	20645313	Liver	348	172	LCP
*Drd1*	20127886	Liver	540	739	LCP

^a^All genes except *Nos1, Map6, Il6* and *Comt* had increased methylation in olanzapine-treated rats.

HCP, high CpG contents; ICP, intermediate CpG contents; LCP, low CpG contents.

Among the genes that showed an increase in methylation in hippocampus was *Bdnf,* which has been previously implicated in schizophrenia [52]. This corroborates previous findings that showed its regulatory role in the expression of the dopamine D3 receptor gene (*DRD3*) [53]*.* The relatively lower methylation and higher expression of *BDNF* was also observed in schizophrenia patients compared to healthy controls [54].

Our results suggested that the efficacy of olanzapine might be achieved by changes in gene-specific methylation of relevant genes that take part in psychosis-related pathways. A list of 123 such genes that increased in methylation in both brain regions is given in Additional file 9: Table S5. That methylation may serve an intermediary role in modulating gene expression is apparent in the cerebellum, which is dominated by a number of signalling pathways including ephrin receptors and synaptic long-term potentiation (Table 2). Ephrin ligands and receptors guide axons during neural development and regulate neuronal plasticity in adults [55,56]. Specifically, ephrin plays an important role in the regulation of neuronal migration, which is essential for the development of the nervous system and the proper functioning of the brain [57]. Neuronal cells have ahigher variation in DNA methylation than non-neuronal cells, supporting the idea that the epigenetic status of neuronal cells changes in response to the environment in the brain [58]. Interestingly, DNA methylation was found to be highly heritable and significantly correlated with gene expression in the human brain [59].

Furthermore, the synaptic long-term potentiation (LTP) pathway was one of the top canonical pathways in the cerebellum. Synaptic activity can persistently modify the way a neuron reacts to subsequent inputs by affecting either its intrinsic excitability or its synaptic efficacy, which is enhanced during long-term potentiation [60]. Specifically, in a rat cerebellum, synaptic transmission and granule cell intrinsic excitability are enhanced during LTP [47]. LTP is a well-known model for synaptic plasticity and it is typically induced by high-frequency activation of NMDA receptors at glutamatergic synapses [61]. Such results allow us to postulate that the efficacy of olanzapine may be due to its effect on the regulation of dopamine*-*DARPP32 feedback in the cAMP signalling pathway in the hippocampus, via DNA methylation.

Further, an atypical antipsychotic induced a restrictive chromatin state in the present study and previous reports [62]. On the other hand, clozapine was found to induce MII1, a mediator of open chromatin [63]. A restrictive chromatin state through DNA methylation has been implicated in psychiatric disorders [64]. Also, olanzapine, unlike clozapine and sulpiride, did not activate brain DNA demethylation in mice [65]. Moreover, atypical antipsychotics might regulate the transcription and function of genes that are related to histone post-transcriptional modifications [62]. Therefore, the mechanisms of actions of olanzapine on the chromatin structure and on any epigenetic machinery need to be studied further.

### Adverse consequences of olanzapine treatment may include aberrant methylation

Although the focus of this study was to assess the effect of olanzapine treatment in the hippocampus and cerebellum, we also used the liver as a non-brain tissue sample. In addition to the pathways implicated in schizophrenia and psychosis, olanzapine treatment also affected pathways for lipid metabolism, metabolic diseases and cell deathin the hippocampus; cardiovascular disease and cell signallingin the cerebellum and cardiovascular system function, cell death and survivalin the liver. These effects may reflect specific adverse consequences such as weight gain [29]. Interestingly, olanzapine has been shown to alterlipid metabolism [65]. Further, *IL6*, which is differentially methylated and is involved in regulating the lipid metabolismpathway, was reported to destabilize atherosclerotic plaques in mice [66]. The *Jak/Stat* signalling pathway, which is affected by olanzapine treatment, is also known to regulate how muscle mass is lost or gained, which is an essential factor in defining obesity [67]. Furthermore, the pathway for the cellular effects of sildenafil (Viagra), which was identified for the hippocampus, may result in sexual dysfunction, which is a common side effect of many antipsychotics [68]. This reflects the possibility that the epigenomic status of the genes involved in this pathway causes this dysfunction.

It is important to point out that the selected pathways discussed are the most significant pathways identified in this study. The study provides a novel insight into the potential mechanisms in the olanzapine-induced reduction of psychosis and the associated adverse effects. Antipsychotics were previously shown to have modulated promoter methylation and thereby gene expression [33,34]. We show for the first time that the pathways affected are for the known effects of olanzapine. Also, the effect of this drug on such pathways may involve alterations in gene-specific methylation. Further, the pathways affected are diverse and tissue specific. Thus, the findings in this report support the involvement of epigenetic changes that are known to be reversible and influenced by genetic as well as environmental factors, in neural function. They offer an original insight into any future epigenetic research in psychiatric disorders and potential avenues for personalized medicine.

## Conclusions

The known functions of affected genes suggest that the observed epigenetic changes may underlie the amelioration of symptoms and account for certain adverse effects including the metabolic syndrome. The results give a novel insight into the mechanism of action of olanzapine, therapeutic effects and the side effects of antipsychotics.

## Methods

### Animals

#### Rats

Adult male Sprague–Dawley rats,12 weeks old weighing 250 to 300 g, were purchased from Charles River, QC, Canada. Upon arrival, the rats were separated into individual cages and housed under controlled humidity and temperature on a 12-hour light/dark cycle (the lights were switched on at 7.00 am). They were fed standard rat chow (LabDiet) and tap water *ad libitum.* The Institutional Animal Care Committee of the University of Western Ontario approved all animal-related procedures used in this study following the Canadian and National Institute of Health Guides on animal experimentation.

#### Olanzapine treatment

Before the commencement of olanzapine treatment, the animals were weighed and divided into two treatment groups with comparable mean weights. They were habituated individually for 30 min to an automated open-field activity-monitoring chamber (San Diego Instruments, San Diego, CA, USA), and then subjected to 5 min of tail pinch stress. Their stress-induced locomotor activity was recorded for the next 30 min using the open-field activity chamber. Starting from the following day, the rats received injections of olanzapine (Zyprexa, Lilly, IN, USA; 2.5 mg/kg, intramuscular; *n* = 8) or vehicle (PBS; *n* = 8) between 1.30 pm and 3.00 pm daily for 19 days. Although antipsychotic drugs have been administered orally and intramuscularly in rodent studies, we chose the intramuscular route to ensure the rats consistently received the intended dose for the entire duration of therapy.

#### Phenotypic analysis and tissue collection

Then 48 hours after the last dose of olanzapine or the vehicle (to avoid olanzapine-induced sedation interfering with stress perception and activity), the rats were habituated to the same open-field activity monitoring chamber for 30 min, subjected to a similar 5-min tail pinch as done at the baseline, and monitored to verify whether stress-induced locomotor activity was reduced in olanzapine-treated rats compared to the vehicle-treated group. This paradigm has been widely used to study the therapeutic efficacy of antipsychotic drugs [27,69]. Subsequently, 24 hours after completion of the stress-induced behavioural assessment (to minimize the potential effect of stress on expected molecular changes), each rat was sacrificed. The rats were decapitated without anaesthesia, brain tissues were micro-dissected promptly in ice-cold PBS and three random biopsy punches through the hippocampus, cerebellum and liver were obtained. These three biopsy punches were considered to be a single sample. The sample from each rat was kept separately and flash-frozen in liquid nitrogen. Genomic DNA was isolated from olanzapine-treated and vehicle-treated samples to analyse the genome-wide methylation using rat methylation arrays.

### Assessing genome-wide DNA methylation by MeDIP-chip analysis

#### Array hybridization

Genomic DNA was isolated from each of the three tissues from two random control samples and two random olanzapine-treated samples. All methylated DNA immunoprecipitation (MeDIP), sample labelling, hybridization, and processing were performed by Arraystar Inc (Rockville, MD, USA). Briefly, isolated genomic DNA was sonicated to generate random fragments of 200 to 1,000 bp. For DNA labelling, the NimbleGen Dual-Color DNA Labeling Kit was used according to the manufacturer’s guideline detailed in the NimbleGen MeDIP-chip protocol (NimbleGen Systems, Inc, Madison, WI, USA). Microarrays were hybridized at 42°C for 16 to 20 h with Cy3/5 labelled DNA in NimbleGen hybridization buffer/hybridization component A in a hybridization chamber (Hybridization System, NimbleGen Systems, Inc, Madison, WI, USA). The methylated DNA was immunoprecipitated using Biomag™ magnetic beads coupled with mouse monoclonal antibodies against 5-methylcytidine. The total input and matched immunoprecipitated DNA were labelled with Cy3- and Cy5-labelled random 9-mers, respectively, and hybridized to NimbleGen RN34 Meth 3×720K CpG plus Promoter arrays. Scanning was performed with the Axon GenePix 4000B microarray scanner.

#### Data normalization and analysis

Raw data was extracted as pair files using the NimbleScan software (Roche NimbleGen Inc). Median-centring quantile normalization and linear smoothing was performed using the Bioconductor packages Ringo, limma, and MEDME. From the normalized log_2_ ratio data, a sliding-window peak-finding algorithm provided by NimbleScan v2.5 (Roche NimbleGenInc) was applied to find the enriched peaks with specified parameters (sliding window width: 750 bp; mini probes per peak: 2; *P-*value minimum cut-off: 2; maximum spacing between nearby probes within peak: 500 bp).

To compare differentially enriched regions between drug-exposed (E) and matched control (C) rats, the log_2_ ratios were averaged and then used to calculate *M*' for each probe:

$$\begin{array}{ll} M'= & \mathrm{Average}\left(\log_{2}\mathrm{MeDIPE}/\mathrm{InputE}\right)\\ & -\mathrm{Average}\left(\log_{2}\mathrm{MeDIPC}/\mathrm{InputC}\right) \end{array}$$

The NimbleScan sliding-window peak-finding algorithm was run on this data to find the differential enrichment peaks (DEPs). The differential enrichment peaks, identified by the NimbleScan algorithm, were filtered according to the following criteria: (i) at least one of the two groups had the median value of log_2_ MeDIP/Input ≥ 0.3 and a median value of *M*' > 0 within the peak; (ii) at least half of the probes in a peak had amedian value of the coefficient of variability (CV) ≤ 0.8 for both groups.

Using an R script program, a hierarchical clustering analysis was completed. The probe data matrix was obtained using PeakScores from differentially methylated regions selected by DEP analysis. This analysis used PeakScore ≥ 2 to define the DEPs, which is equivalent to the average *P* ≤ 0.01, for all probes within the peak.

#### Pathway and bioinformatic analysis of array results

A venn diagram of the genes was used to assess the distribution of genes affected across tissue types [70]. The identified gene promoters with significant alterations to DNA methylation were then subjected to Ingenuity Pathway Analysis (Ingenuity System Inc, CA, USA) [71].

## Abbreviations

Bp: Base pair; CV: Coefficient of variability; DEP: Differential enrichment peak; HCP: High CpG contents; ICP: Intermediate CpG contents; LCP: Low CpG contents; LTP: Long-term potentiation; MeDIP: Methylated DNA immunoprecipitation; PBS: Phosphate-buffered saline.

## Competing interests

The authors declare that they have no competing interests.

## Authors’ contributions

MGM analyze the data and wrote the first draft of the manuscript. BIL helped analyze the data and reviewed the manuscript. PM worked on genomic DNA extraction from the rat brain regions and liver. CAC critically reviewed the manuscript for important intellectual content. NR prepared the animals and provided animal tissues. NR and RO participated in the design of the study and also critically reviewed the manuscript for important intellectual content. SMS conceived of the study and critically reviewed and approved the manuscript. All authors have approved the final version of the manuscript.

## Supplementary Material

Additional file 1 Supplementary figures. Click here for file

Additional file 2: Table S1A Genes with increased methylation in the hippocampus as a result of olanzapine treatment. Click here for file

Additional file 3: Table S1B Genes with decreased methylation in the hippocampus as a result of olanzapine treatment. Click here for file

Additional file 4: Table S2A Genes with increased methylation in the cerebellum as a result of olanzapine treatment. Click here for file

Additional file 5: Table S2B Genes with decreased methylation in thecerebellum as a result of olanzapine treatment. Click here for file

Additional file 6: Table S3A Genes with increased methylation in the liver as a result of olanzapine treatment. Click here for file

Additional file 7: Table S3B Genes with decreased methylation in the liver as a result of olanzapine treatment. Click here for file

Additional file 8: Table S4 Most significant networks identified by pathway analysis for genes that had **(a)** an increase or **(b)** a decrease in methylation, in the liver following olanzapine treatment. Click here for file

Additional file 9: Table S5 Genes with increased methylation in both the hippocampus and cerebellum following olanzapine treatment. Click here for file
