# Supplementary material for: The effects of olanzapine on genome-wide DNA methylation in the hippocampus and cerebellum
Source: Clin Epigenetics. 2014 Jan 2;6(1):1. doi: 10.1186/1868-7083-6-1 (PMC3895844; doi:10.1186/1868-7083-6-1)
Supplement: Additional file 1 — Supplementary figures. [file 1868-7083-6-1-S1.doc]

**Supplementary Figure Legends**

**Suppl. Fig. 1.** Baseline (no stress) and post-stress locomotor activity (a) and body weight (b) in olanzapine treated and control rats, assessed on day 0 and day 21 (48 hours after the last treatment of olanzapine). Olanzapine treatment started at day 1. Olanzapine treated and control rats showed a 10.7% and 2.7% increase in body weight from day 0 to day 21, respectively (It represents a fourfold increase in weight in drug treated rats as compared to controls). The corresponding decrease in locomotor activity in baseline (no stress) was 23.2% and 22.3%, respectively; while post-stress it was reduced by 65.6% and 15.6%, respectively (It represents 4.2% reduction in locomotor activity in drug treated rats).

**Suppl. Fig. 2**. Metabolic Disease, Tissue Morphology, Endocrine System Disorders network involving genes with increased methylation, in hippocampus. The stars indicate genes previously implicated in schizophrenia (From Ingenuity Pathway Analysis).

**Suppl. Fig. 3**. *Cellular Effects of Sildenafil (Viagra) pathway* involving the 127 genes that were differentially methylated in hippocampus and cerebellum as a result of olanzapine treatment (From Ingenuity Pathway Analysis).

**Suppl. Fig. 4**. Synaptic Long Term Potentiation was among the top canonical pathways involving genes with increased methylation, in cerebellum, following olanzapine treatment (From Ingenuity Pathway Analysis).

**Suppl. Fig. 5.** Tissue Morphology, Cellular Assembly and Organization, Nervous System Development and Function were among the top networks involving genes with increased methylation, in liver. The stars indicate genes previously implicated in schizophrenia (From Ingenuity Pathway Analysis).

**Suppl. Fig. 6**. *Molecular Transport, Cell-To-Cell Signaling and Interaction, Nervous System Development and Function* network involving genes with reduced methylation, in hippocampus, following olanzapine treatment. The star indicates a gene previously implicated in schizophrenia (From Ingenuity Pathway Analysis).


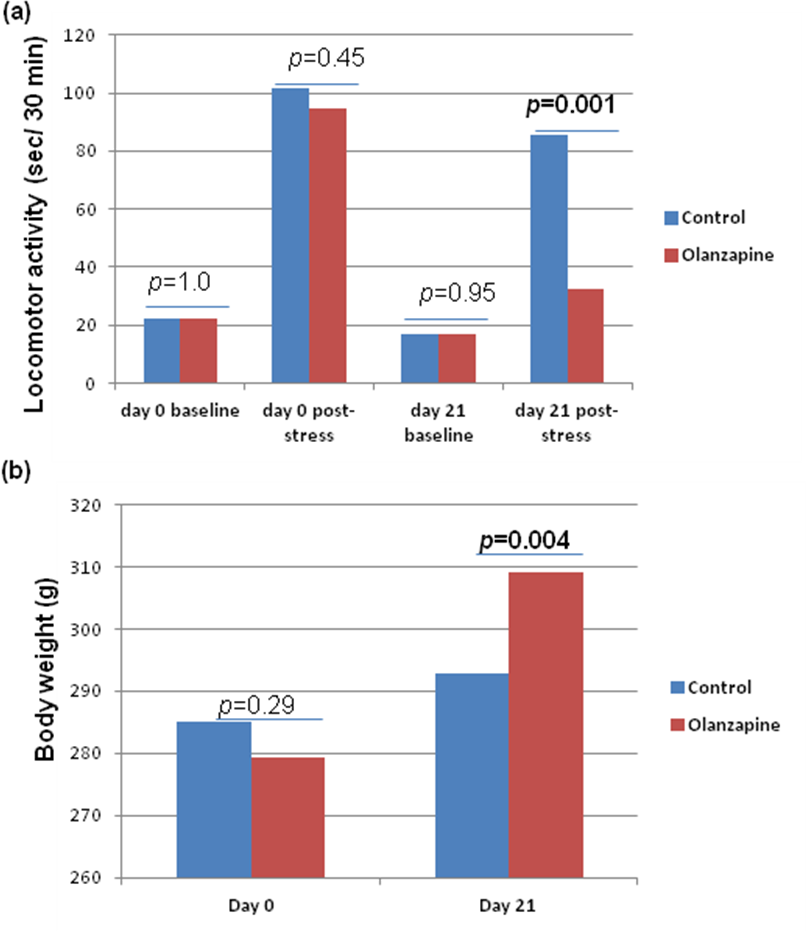


**Suppl. Fig. 1**. Baseline and post-stress locomotor activity (a) and body weight (b) in olanzapine treated and control rats, assessed on day 0 and day 21.


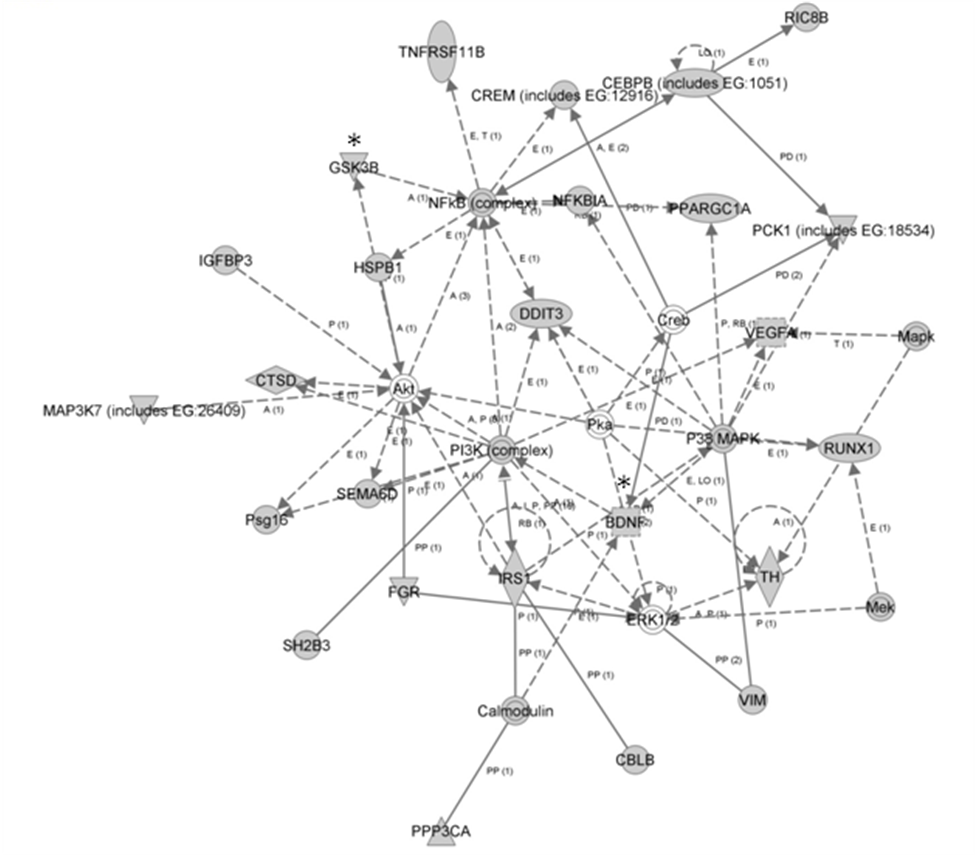


**Suppl. Fig. 2**. Metabolic Disease, Tissue Morphology, Endocrine System Disorders.


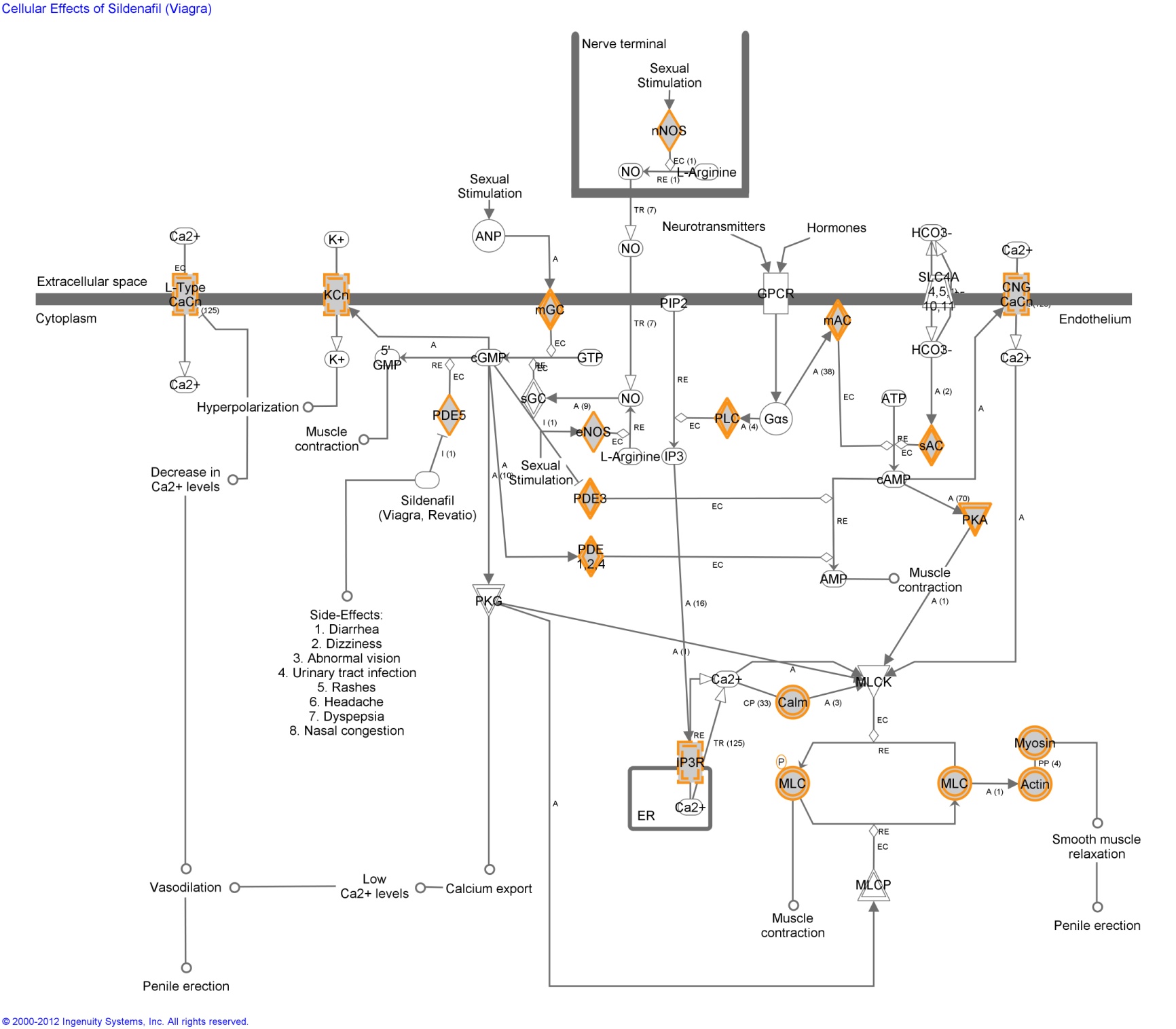


**Supple. Fig. 3.** Cellular Effects of *Sildenafil* (*Viagra*) pathway.


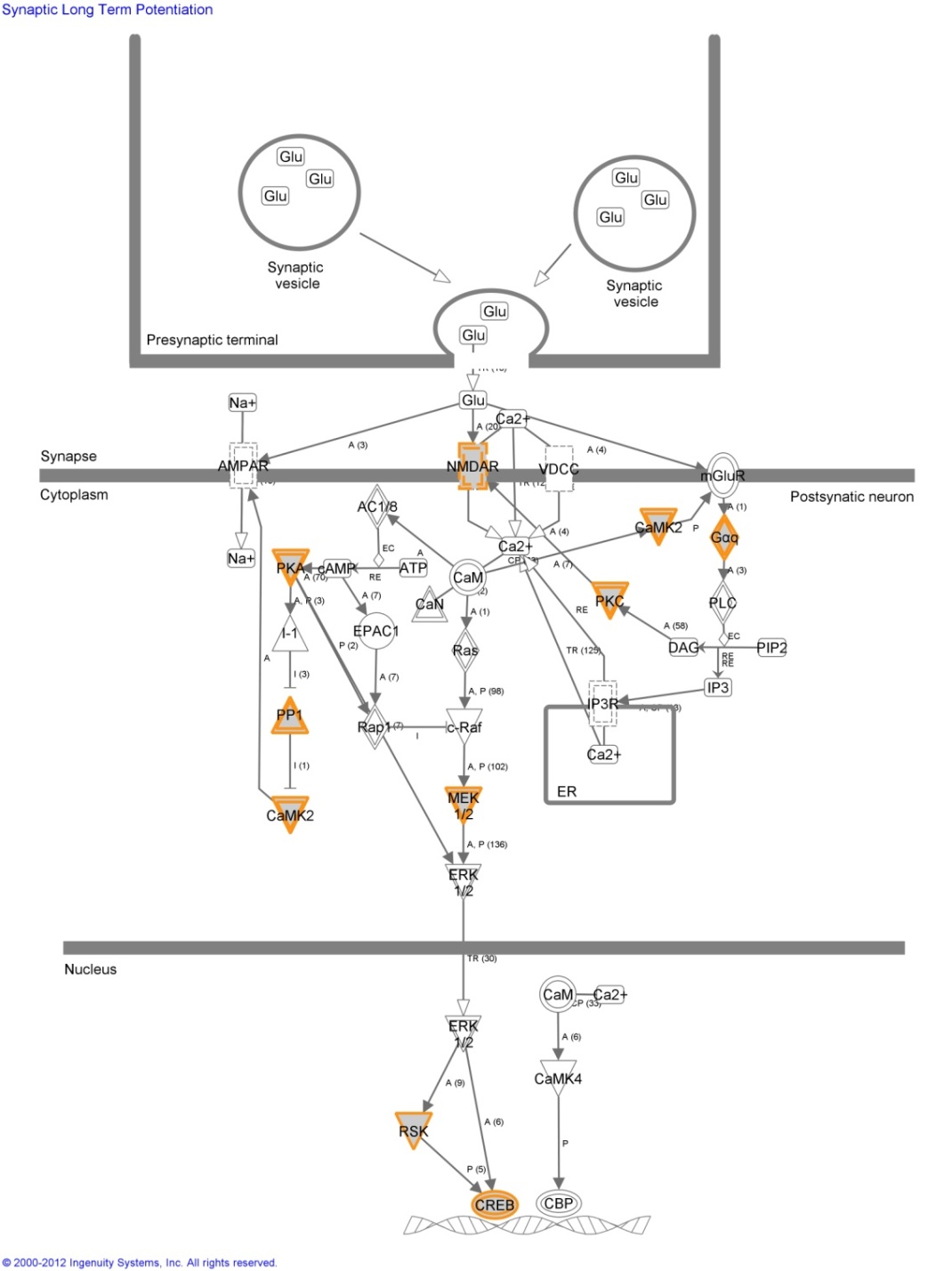


**Suppl. Fig. 4**. Synaptic Long Term Potentiation pathway.


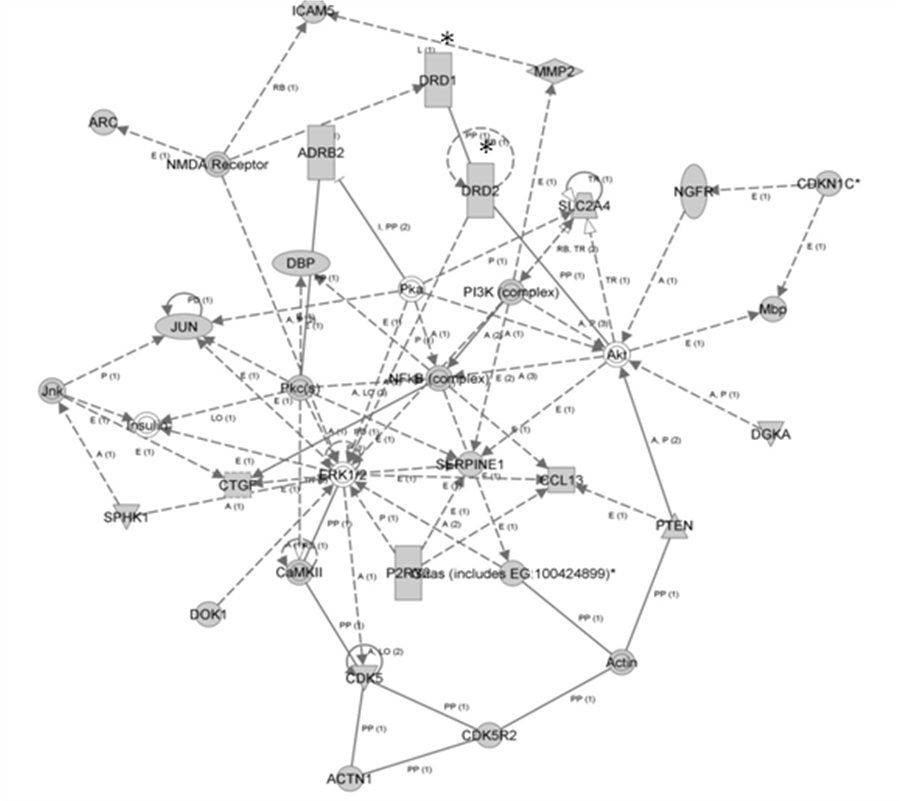


**Suppl. Fig. 5**. Tissue Morphology, Cellular Assembly and Organization, Nervous System Development and Function


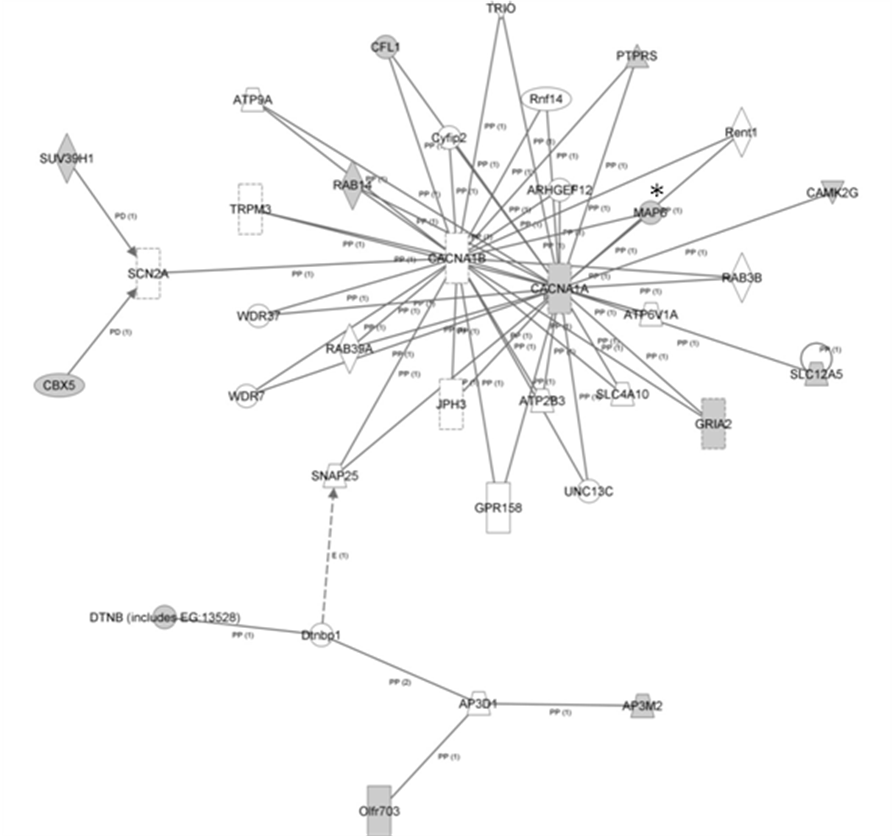


**Suppl. Fig. 6**. Molecular Transport, Cell-To-Cell Signaling and Interaction, Nervous System Development and Function
