# Supplementary material for: The effects of olanzapine on genome-wide DNA methylation in the hippocampus and cerebellum
Source: Clin Epigenetics. 2014 Jan 2;6(1):1. doi: 10.1186/1868-7083-6-1 (PMC3895844; doi:10.1186/1868-7083-6-1)
Supplement: Additional file 8: Table S4 — Most significant networks identified by pathway analysis for genes that had (a) an increase or (b) a decrease in methylation, in the liver following olanzapine treatment. [file 1868-7083-6-1-S8.doc]

**Table S4. Most significant networks identified by pathway analysis using genes that showed (a) an increase and (b) a decrease in methylation, in liver, following Olanzapine treatment**

| **(a) Top Canonical pathways** | **p-value** | **# Molecules^1^** |
| --- | --- | --- |
| Gα12/13 Signaling | 7.24E-04 | 18/116 (0.155) |
| Glucocorticoid Receptor Signaling | 1.39E-03 | 31/258 (0.12) |
| Estrogen Receptor Signaling | 1.69E-03 | 18/122 (0.148) |
| G-Protein Coupled Receptor Signaling | 2.32E-03 | 49/489 (0.1) |
| eNOS Signaling | 2.49E-03 | 18/124 (0.145) |
| **Associated Network Functions** |  |  |
| Cardiovascular System Development and Function, Cell Death and Survival, Organ Morphology, Nervous System Development and Function | | 42 |
| Neurological Disease, Organismal Injury and Abnormalities, Tissue Morphology | | 12 |
| Carbohydrate Metabolism, Cancer, Developmental Disorder | | 10 |
| Cell Death and Survival, Connective Tissue Development and Function, Skeletal and Muscular System Development and Function | | 10 |
| **(b) Top Canonical Pathways** | **p-value** | **# Molecules** |
| Acute Phase Response Signaling | 2.96E-03 | 12/164 (0.073) |
| CDP-diacylglycerol Biosynthesis I | 5.96E-03 | 3/15 (0.2) |
| Clathrin-mediated Endocytosis Signaling | 1.27E-02 | 11/176 (0.062) |
| JAK/Stat Signaling | 1.44E-02 | 6/68 (0.088) |
| Calcium Transport I | 2.19E-02 | 2/9 (0.222) |
| **Associated Network Functions** |  |  |
| Lipid Metabolism, Small Molecule Biochemistry, Cell Death and Survival | | 29 |
| Cell-To-Cell Signaling and Interaction, Nervous System Development and Function, Molecular Transport | | 14 |
| Cancer, Endocrine System Disorders, Cell Cycle | | 12 |
| Cellular Development, Cellular Growth and Proliferation, Tumor | | 9 |
| Lipid Metabolism, Molecular Transport, Small Molecule Biochemistry | | 6 |

^1^The number of molecules for the top canonical pathways is presented as a ratio of molecules that meet the cut-off (p<=0.01) for the significance threshold over all the molecules involved in the pathway.
